# Do patients with femoroacetabular impingement syndrome who undergo hip arthroscopy display improved alpha angle (magnetic resonance imaging) and radiographic hip morphology?

**DOI:** 10.1111/1756-185X.14530

**Published:** 2022-12-11

**Authors:** Aricia Jieqi Thirumaran, Nicholas J Murphy, Jillian Peta Eyles, James M. Linklater, Stephan Reichenbach, Florian Schmaranzer, Till D. Lerch, Venkatesha Venkatesha, Gillian Heller, John O'Donnell, David J. Hunter

**Affiliations:** ^1^ Faculty of Medicine and Health University of New South Wales Sydney New South Wales Australia; ^2^ Sydney Musculoskeletal Health, Kolling Institute of Medical Research University of Sydney Camperdown New South Wales Australia; ^3^ Department of Orthopedic Surgery John Hunter Hospital New Lambton New South Wales Australia; ^4^ Department of Rheumatology Royal North Shore Hospital Sydney New South Wales Australia; ^5^ Department of Musculoskeletal Imaging Castlereagh Imaging St Leonards New South Wales Australia; ^6^ Institute of Social and Preventive Medicine University of Bern Bern Switzerland; ^7^ Department of Rheumatology, Immunology and Allergology University Hospital and University of Bern Bern Switzerland; ^8^ Department of Diagnostic, Interventional and Pediatric Radiology, Inselspital Bern University Hospital, University of Bern Bern Switzerland; ^9^ Northern Sydney Local Health District Executive Royal North Shore Hospital St Leonards New South Wales Australia; ^10^ Department of Statistics, Faculty of Science and Engineering Macquarie University Sydney New South Wales Australia; ^11^ Hip Arthroscopy Australia Melbourne Victoria Australia; ^12^ St Vincent's Private Hospital Melbourne Victoria Australia

**Keywords:** arthroscopic surgery, computer assisted radiographic image interpretation, femoroacetabular impingement, hip osteoarthritis, physiotherapy

## Abstract

**Aims:**

To compare (a) the change in radiological bony morphology between participants with femoroacetabular impingement (FAI) syndrome who underwent arthroscopic hip surgery compared to physiotherapist‐led non‐surgical care and (b) the change in radiological bony morphology between participants with FAI syndrome who underwent arthroscopic hip surgery involving cam resection or acetabular rim trimming or combined cam resection and acetabular rim trimming.

**Methods:**

Maximum alpha angle measurements on magnetic resonance imaging and Hip^2^Norm standardized hip measurements on radiographs were recorded at baseline and at 12 months postoperatively. One‐way analysis of covariance and independent *T* tests were conducted between participants who underwent arthroscopic hip surgery and physiotherapist‐led non‐surgical care. Independent *T* tests and analysis of variance were conducted between participants who underwent the 3 different arthroscopic hip procedures.

**Results:**

Arthroscopic hip surgery resulted in significant improvements to mean alpha angle measurements (decreased from 70.8° to 62.1°) (*P* value < .001, 95% CI −11.776, −4.772), lateral center edge angle (LCEA) (*P* value = .030, 95% CI −3.403, −0.180) and extrusion index (*P* value = 0.002, 95% CI 0.882, 3.968) compared to physiotherapist‐led management. Mean maximum 1‐year postoperative alpha angle was 59.0° (*P* value = .003, 95% CI 4.845, 18.768) for participants who underwent isolated cam resection. Measurements comparing the 3 different arthroscopic hip procedures only differed in total femoral head coverage (F[2,37] = 3.470, *P* = .042).

**Conclusion:**

Arthroscopic hip surgery resulted in statistically significant improvements to LCEA, extrusion index and alpha angle as compared to physiotherapist‐led management. Measured outcomes between participants who underwent cam resection and/or acetabular rim trimming only differed in total femoral head coverage.

## 1 INTRODUCTION

Femoroacetabular impingement (FAI) syndrome^1^ is characterized by abnormal contact between an aspherical femoral head and acetabular rim during hip flexion,^2^ leading to cartilage surface damage and labral detachment.^2^, ^3^ This leads to pain and early structural damage,^4^ which are thought to lead to osteoarthritis of the hip.^3^, ^5^, ^6^ Cam‐type FAI syndrome is characterized by the flattening of the femoral head and neck junction while pincer‐type FAI syndrome is characterized by excessive coverage of the femoral head by the acetabulum.^7^

To assess hip shape in individuals with FAI syndrome, anteroposterior, lateral pelvic and Dunn view radiographs are taken. Magnetic resonance imaging (MRI) arthrography enables the visualization of damage to either the labrum or cartilage.^8^ However, due to patient position, differences in pelvic orientation while in supine and standing position, and rotation of legs, it is difficult to assess and compare acetabular morphology before and after interventions. Current techniques to correct for this variation are imprecise and inaccurately represent hip morphology, resulting in inappropriate surgical recommendations and unsuitable resections of the acetabulum.^9^ Additionally, standardization is required as variation in pelvic tilt can lead to a falsely diagnosed retroverted acetabulum.^10^ Hence, Hip^2^Norm was developed to standardize measurements of acetabular morphology on anteroposterior radiographs, correcting for patient pelvic tilt and rotation.^11^ To calculate hip parameters using Hip^2^Norm, a combination of lateral pelvic and anteroposterior views are required, while minimizing movement or changes to position.^8^

The alpha angle has also been used to define and quantify cam morphology in FAI.^12^, ^13^ It represents the angle between the femoral head and neck junction, using the angle between the anterior point where the center of the head of the femur exceeds the radius of the femoral head's subchondral surface and the narrowest point of the center of the neck of femur.^14^ The larger the alpha angle, the greater the risk of osteoarthritis and total hip replacement.^15^

This study aimed to determine the presence of a change in radiological bony morphology of participants with FAI syndrome following arthroscopic hip surgery using Hip^2^Norm standardized hip measurements^11^ and alpha angle measurements as well as to compare change in radiological bony morphology between participants with FAI syndrome who underwent arthroscopic hip surgery involving cam resection or acetabular rim trimming or combined cam resection and acetabular rim trimming.

## 2 METHODS

This study involved secondary analyses of data collected from the Australian FASHIoN trial, a multi‐center randomized controlled trial, comparing 12‐month hip cartilage health between participants who underwent arthroscopic hip surgery and physiotherapist‐led non‐surgical care for the treatment of FAI syndrome.^12^ The trial's primary outcome was the change in delayed gadolinium‐enhanced MRI of cartilage (dGEMRIC) index between baseline and postoperative 12 months.^12^

Inclusion criteria for participation in the study were: age 16 years or over, symptomatic hip pain (including clicking, catching or giving way), radiological signs of FAI syndrome (alpha angle >55° for cam morphology, lateral center edge angle [LCEA] >40° or other radiographic signs of pincer morphology such as positive cross‐over sign) and the treating orthopedic surgeon being of the opinion that the patient would benefit from arthroscopic surgery for FAI syndrome. Ninety‐nine participants were recruited from private or public clinics of 8 orthopedic surgeons with a 1:1 allocation ratio.^12^

Participants were randomized to arthroscopic hip surgery or physiotherapist‐led non‐surgical care, called personalized hip therapy (PHT). PHT was delivered for a maximum of 6 months from the date of randomization, with 6 compulsory sessions within the first 12 weeks and a maximum of 10 sessions. Participants allocated to the arthroscopic hip surgery underwent surgery no more than 18 weeks after treatment allocation.^12^

PHT involved individualized, progressive exercise rehabilitation with adjunctive anti‐inflammatory medications and/or intra‐articular corticosteroid injections. Arthroscopic hip surgery was performed under general anesthesia and involved the resection of deformities at the acetabular rim and head–neck junction, while using intra‐operative radiography, and ensuring that hip range of motion was acceptable. Repair of the acetabular labrum and cartilage damage was undertaken where necessary. Participants were discharged from hospital when able to mobilize safely with crutches and their postoperative rehabilitation was dependent on their surgeon's rehabilitation protocol.^12^

## 3 OUTCOMES

Outcome data were collected post‐randomization at baseline and postoperative 12 months. Participants underwent a supine abdominal projection, Dunn view plain radiograph and MRI scan of the pelvis at baseline and at postoperative 12 months.

### 3.1 Radiographic assessment

Hip^2^Norm was used for standardization of hip radiographic parameters for participants in both intervention groups at baseline and at postoperative 12 months. Hip^2^Norm corrects for pelvic tilt and hip rotation, allowing standardization and accurate calculation of radiographic parameters of hip morphology.^11^ Hip^2^Norm parameters are defined in Table S1.^11^

### 3.2 Alpha angle measurements

Alpha angle measurements were taken (see Figure S1), from anterior to superior, at 30‐degree intervals, on preoperative and 12‐month postoperative radially reformatted MRI scans. Measurements were made by a trained reader (NM), and intra‐ and inter‐rater reliability intraclass coefficients (ICCs) were measured and found to be good (intra‐rater ICC 0.89, 95% CI: 0.84‐0.93; inter‐rater ICC, 0.89 95% CI: 0.83‐0.93), as previously reported.^16^ The maximum of the alpha angles measured in the radial planes for each participant was reported.

## 4 STATISTICAL ANALYSIS

Statistical analyses were conducted in SPSS (Version 26, Armonk NY: IBM Corp). Mean differences in each hip radiographic parameter were compared at baseline and postoperative 12‐month within each intervention group and calculated with a 95% CI. The mean difference of hip parameters after Hip^2^Norm standardization at baseline and postoperative 12 months were compared between the PHT and hip arthroscopy groups using an unadjusted independent *T* test. A one‐way analysis of covariance (ANCOVA) was used to compare the mean change between the 2 intervention groups while adjusting for the baseline value. Robust standard errors were used for parameters found to be statistically significant for heteroskedasticity using the modified Breusch‐Pagan test. The same approach was conducted for alpha angle measurements of both intervention groups.

Additionally, the mean difference of hip parameters after Hip^2^Norm standardization and alpha angle at baseline and postoperative 12 months were compared within hip arthroscopic procedure subgroups (cam resection only or acetabular rim trimming only or both cam resection and acetabular rim trimming) using an unadjusted independent *T* test. Cohen's d was calculated for parameters which were statistically significant. A Cohen's d value of 0.2, 0.5 and 0.8 indicate a small, medium and large effect size, respectively.^17^ McNemar's test was done in place of an unadjusted independent *T* test for cross‐over sign and posterior wall sign. An analysis of variance (ANOVA) was used to compare the mean change between the 3 subgroups. The same approach was conducted for alpha angle measurements.

## 5 RESULTS

The study sample consisted of 99 participants with a mean age of 32.9 years. There were 57.6% of participants who were male, and the mean height and body mass index of the sample was 175.6 cm and 24.3 kg/m^2^, respectively. Mean age at onset of symptoms was at 30.9 years and 20% of participants had bilateral symptoms (see Table S2). Within participants who had arthroscopic hip surgery, 41.2% who had a cam resection only and 45.5% who had both cam resection and acetabular rim trimming had residual cam morphology. However, all participants who only had an acetabular rim trimming had residual cam morphology.

### 5.1 Radiographic parameters

There were no significant differences in radiographic parameters when comparing participants who underwent PHT and arthroscopic hip surgery except for LCEA (*P* value = .030, 95% CI −3.403, −0.180) and extrusion index (*P* value = .002, 95% CI 0.882, 3.968) which favored arthroscopic hip surgery (see Table S3). There was a mean decrease of 2.90° in LCEA within participants who underwent arthroscopic hip surgery as compared to 0.41° in participants who underwent PHT. The change in extrusion index indicated a significant increase in percentage of uncovered femoral head as compared to the total horizontal head diameter of the femur.

Within the participants who underwent arthroscopic hip surgery, there was a statistically significant improvement in the acetabulum (ACM) angle (*P* value = .001, 95% CI 0.524, 1.703, Cohen's d = 1.05) between baseline and 12‐month postoperative radiographic measurements in participants who underwent a cam resection only. Participants who underwent both cam resection and acetabular rim trimming had statistically significant improvements in total femoral head coverage (*P* value < .001, 95% CI 3.360, 8.100, Cohen's d = 1.13), LCEA (*P* value < .001, 95% CI 2.649, 6.571, Cohen's d = 1.10), acetabular index (*P* value = .047, 95% CI −3.770, −0.0297, Cohen's d = 0.475) and extrusion index (*P* value <.001, 95% CI −6.702, −3.138, Cohen's d = 1.29) between baseline and 12‐month postoperative radiographic measurements. There was no statistically significant difference between baseline and 12‐month postoperative measurements in participants who underwent acetabular rim trimming only. There is no statistically significant difference in standardized measurements except for total femoral head coverage (F[2,37] = 3.470, *P* = .042) when comparing the 3 subgroups (see Table S4).

*P* value of McNemar's test on cross‐over sign in cam resection only and both cam resection and acetabular rim trimming had a value of 1.000 and .625, respectively. *P* value of McNemar's test on posterior wall sign in cam resection only and both cam resection and acetabular rim trimming had a value of 1.000 and 1.000, respectively (see Table S4). These results are statistically insignificant and indicate there was no difference between the baseline and 12‐month postoperative measurements for cross‐over sign and posterior wall sign in the cam resection only and both cam resection and acetabular rim trimming groups. The sample size of the acetabular rim trimming only group was too small to conduct a McNemar's test and there was no variation in measurements from baseline to postoperative 12 months.

### 5.2 Alpha angle

Arthroscopic hip surgery resulted in statistically significant decreases in alpha angle measurements on radially reformatted MRI scans, from 70.8° to 62.1° (*P* value < .001, 95% CI −11.776, −4.772) (see Table S3). The correlation between a change in alpha angle and a change in International Hip Outcome Tool (iHOT)‐33 score at 1 year was weak and not statistically significant in both PHT (Pearson correlation = 0.004, *P* value = .980) and arthroscopic surgery (Pearson correlation = 0.056, *P* value = .721) intervention groups (see Table S5).

Prevalence of postoperative alpha angle >55° in participants who underwent arthroscopic surgery or PHT at 12‐month follow‐up was 55.8% and 78.0%, respectively. Prevalence of postoperative alpha angle >60° in participants who underwent arthroscopic surgery or PHT at 1‐year follow‐up was 41.9% and 58.5%, respectively.

Within participants who underwent arthroscopic hip surgery, there was a statistically significant improvement in the alpha angle measurements (*P* value = .003, 95% CI 4.845, 18.768, Cohen's d = 0.939) in participants who underwent a cam resection only with a mean maximum postoperative alpha angle of 59.0° at 12‐month follow‐up. Prevalence of postoperative alpha angle >55° in participants who underwent cam resections only at 12‐month follow‐up was 46.7%. Participants who underwent both cam resection and acetabular rim trimming had a statistically significant improvement in alpha angle measurements (*P* value = .002, 95% CI 3.938, 14.528, Cohen's d = 0.794). There was no statistically significant difference in alpha angle measurements when comparing the 3 subgroups (F[2,38] = 1.713, *P* = .194) (see Table S4).

## 6 DISCUSSION

As expected, this study demonstrates significant improvements in LCEA, extrusion index and alpha angle measurements of participants who underwent arthroscopic hip surgery. Similarly, Haefeli et al^18^ found a significant decrease in postoperative LCEA (*P* < .001) and alpha angle (*P* < .001). However, the increase in extrusion index of participants in this study was not significant (*P* = .107).^18^ Haug et al^19^ also found a statistically significant decrease in alpha angle measurements of post‐arthroscopy patients.^19^

LCEA is associated with acetabular over‐coverage and values greater than 39° indicate pincer‐type impingement.^8^ The mean values of LCEA of participants in both intervention groups were above the normal range of 23° and 33°^20^ prior to the intervention. This study showed a mean difference of −2.9° LCEA of participants who underwent arthroscopic hip surgery as compared to 0.41° in participants who underwent PHT. This brought the mean LCEA of participants who underwent arthroscopic surgery closer to the normal range, indicating that arthroscopic surgery reduces over‐coverage. However, studies have shown that an elevated LCEA has been observed to be associated with risk of requiring revision surgery.^18^, ^21^

Femoral head extrusion index indicates the percentage of uncovered femoral head, with a normal range of 17%‐27%.^20^ The mean values of extrusion index of participants in PHT were within the normal range prior to the interventions. However, the mean values of the extrusion index of participants who underwent arthroscopic hip surgery improved from 15% to 18.2%, leading to a 3.29% increase as compared to a 0.17% increase in participants who underwent PHT. This indicates that arthroscopic surgery was successful at reducing over‐coverage.

Abnormal LCEA and extrusion index values are associated with pincer‐type hip impingements.^8^ Our results indicate that arthroscopic hip surgery may have improved outcomes for participants with pincer‐type hip impingements as compared to cam‐type hip impingements, possibly due to differences in morphology.

Alpha angle measurements represent the angle between the femoral head and neck junction^14^ and a threshold of 55° is considered abnormal in this study. Despite the significant reduction in mean alpha angle associated with arthroscopic hip surgery of 8.8° (see Table S3), the 12‐month postoperative mean alpha angle was 62.1° which is still above our threshold that indicates a residual cam lesion. There were 55.8% of participants who had postoperative alpha angle measurements greater than 55° following arthroscopic hip surgery, indicating residual cam deformities. As greater alpha angle values are associated with reduced internal and external rotation with hip flexion and hip abduction,^22^ the reduction in alpha angle measurement following arthroscopic hip surgery may indicate an improvement in participants' range of motion.

Participants who underwent both cam resection and acetabular rim trimming had significant improvements in multiple Hip^2^Norm and alpha angle measurements from baseline while participants who underwent a cam resection only saw improvements in ACM angle and alpha angle measurements from baseline. Participants who underwent acetabular rim trimmings saw little improvement in their measurements. This could possibly be explained by the small number of participants within the acetabular rim trimming only group. However, our study found no difference in alpha angle measurements and Hip^2^Norm measurements between participants who underwent cam resection and/or acetabular rim trimming except for total femoral head coverage. This indicates that having both cam resection and acetabular rim trimming may result in superior reductions to total femoral head coverage and may be beneficial for participants with a pincer‐type FAI syndrome which is characterized by an over‐coverage of the femur.

Taking into consideration the primary analyses by Hunter et al^23^ who found no statistically significant difference in cartilage health based on dGEMRIC scores between participants who underwent arthroscopy as compared to PHT, this study reveals that although there may be a physical change in the anatomy of the hip joint post‐surgery, the quality of the cartilage and subsequent risk of osteoarthritis in the future may not differ between comparison groups at 12 months. Additionally, given the weak correlation between the change in alpha angle measurements and iHOT‐33 scores, an improvement in alpha angle measurements does not indicate an improvement in the reported quality of life by participants at 12 months. Hence, these radiological changes may have demonstrated limited clinical relevance at 12 months. However, long term studies are necessary to determine the continuing impact of changes to alpha angle on participant quality of life and future risk of osteoarthritis.

### 6.1 Limitations

Lateral pelvic X‐rays of participants were not taken in this study. This is needed to calibrate the individual pelvic tilt by measuring the vertical distance from sacrococcygeal joint and superior border of public symphysis.^8^ However, Tannast et al^24^ found that LCEA, acetabular index, extrusion index and ACM angle have no significant variation with pelvic tilt and rotation.^24^ Additionally, a femoral version parameter on MRI was not included. Reduced femoral version is seen in cam deformities and has been associated with anterior extra‐articular hip impingement.^25^, ^26^ We were also unable to compare the degree of anatomical change to changes in participants' range of motion as these data were not systematically collected. Although increasing the cut‐off threshold from 55° to 60° would decrease the prevalence of participants with persisting cam lesions, further information regarding participant clinical outcomes is required for analysis of its clinical significance.

We acknowledge the controversy in alpha angle measurements regarding their appropriate threshold for FAI syndrome. While a higher threshold of 60° may increase specificity^27^ and has also been suggested by a systematic review by van Klij et al,^28^ we chose 55° as our threshold to be consistent with the UK FASHIoN randomized controlled trial.^7^ As this was an exploratory analysis of the relationship between surgical or non‐surgical managements of FAI syndrome and hip morphology, we did not include a Bonferroni correction despite having multiple comparisons.

## 7 CONCLUSION

In summary, this study concludes that arthroscopic hip surgery results in superior improvements to LCEA, extrusion index and alpha angle measurements in participants as compared to PHT. We also found no difference in measurement outcomes between the different types of surgical procedures apart from total femoral head coverage which had significant reductions in measurements when both cam resection and acetabular rim trimming were performed.

## CONFLICT OF INTEREST

David J Hunter is a consultant to Pfizer, Lilly, TLCBio and Merck Serono and is supported by a National Health and Medical Research Council Practitioner Fellowship. The other authors do not have any competing interests.

## Supporting information


Table S1.

Table S2.

Table S3.

Table S4.

Table S5.

Figure S1.
