# Supplementary material for: Do patients with femoroacetabular impingement syndrome who undergo hip arthroscopy display improved alpha angle (magnetic resonance imaging) and radiographic hip morphology?
Source: Int J Rheum Dis. 2022 Dec 11;26(2):354–9. doi: 10.1111/1756-185X.14530 (PMC10946938; doi:10.1111/1756-185X.14530)
Supplement: Supplementary file 1 — Table S1. [file APL-26-354-s006.docx]

**Supplementary Tables**

**Table 1:** Definitions of Hip^2^Norm evaluated parameters^1^.

| Parameters | Definition |
| --- | --- |
| Anterior coverage | Percentage of femoral head covered by the acetabulum in antero-posterior direction |
| Posterior coverage | Percentage of femoral head covered by the acetabulum in postero-anterior direction |
| Total femoral head coverage | Percentage of femoral head covered by the acetabulum in cranio-caudal direction |
| Lateral center edge angle (LCEA) | Angle between a line parallel to the longitudinal pelvic axis and a line between the center of the femoral head and lateral edge of the acetabular sourcil |
| Acetabular index | Angle between by a horizontal line and a tangent from the lowest point of the sclerotic zone of the acetabular roof to the lateral edge of the acetabular sourcil |
| Acetabular center-margin (ACM) angle | Angle constructed by:   - The superolateral acetabular edge - The midpoint of a line connecting the superolateral and inferolateral acetabular edge - The point of the bony acetabulum intersected by a perpendicular line relative to the line between the superolateral acetabular edge and midpoint of the superolateral and inferolateral acetabular edge, through the midpoint of a line connecting the superolateral and inferolateral acetabular edge |
| Extrusion index | Percentage of uncovered femoral head in comparison to the total horizontal head diameter |
| Cross-over sign | Positive if anterior acetabular rim is projected more laterally than the posterior rim in the cranial part of the acetabulum |

**References**

1. Tannast M, Mistry S, Steppacher SD, et al. Radiographic analysis of femoroacetabular impingement with Hip2Norm-reliable and validated. *J Orthop Res* 2008; 26: 1199-1205. 2008/04/12. DOI: 10.1002/jor.20653.
