# Supplementary material for: Do patients with femoroacetabular impingement syndrome who undergo hip arthroscopy display improved alpha angle (magnetic resonance imaging) and radiographic hip morphology?
Source: Int J Rheum Dis. 2022 Dec 11;26(2):354–9. doi: 10.1111/1756-185X.14530 (PMC10946938; doi:10.1111/1756-185X.14530)
Supplement: Supplementary file 2 — Table S2. [file APL-26-354-s002.docx]

**Table 2:** Participant demographics and baseline characteristics, reported as mean (standard deviation)

| Parameter | Overall (n=99) | Physiotherapist-led non-surgical care (n= 50) | Arthroscopic hip surgery (n= 49) |
| --- | --- | --- | --- |
| Age (years) | 32.9 (10.5) | 32.9 (9.1) | 32.9 (11.8) |
| Male, n (%) | 57 (57.6) | 26 (52.0) | 31 (63.3) |
| Height (cm) | 175.6 (8.6) | 174.9 (8.9) | 176.3 (8.3) |
| Body mass (kg) | 75.3 (13.0) | 74.9 (12.2) | 75.7 (13.8) |
| Body mass index (kg/m^2^) | 24.3 (3.0) | 24.4 (2.6) | 24.2 (3.5) |
| Age of onset of symptoms (years) | 30.9 (10.8) | 30.2 (9.3) | 31.6 (12.1) |
| Presence of bilateral symptoms, n (%) | 20 (20.2) | 11 (22.0) | 9 (18.4) |
| Private patients, n (%) | 39 (39.4) | 20 (40.0) | 19 (38.8) |
| Surgical procedure done | | | |
| Neither cam resection nor acetabular rim trimming |  |  | 2 |
| Cam resection only |  |  | 17 |
| Acetabular rim trimming only |  |  | 5 |
| Both cam resection and acetabular rim trimming |  |  | 22 |
