# Supplementary material for: Do patients with femoroacetabular impingement syndrome who undergo hip arthroscopy display improved alpha angle (magnetic resonance imaging) and radiographic hip morphology?
Source: Int J Rheum Dis. 2022 Dec 11;26(2):354–9. doi: 10.1111/1756-185X.14530 (PMC10946938; doi:10.1111/1756-185X.14530)
Supplement: Supplementary file 3 — Table S3. [file APL-26-354-s004.docx]

**Table 3:** Adjusted comparison of Hip^2^Norm measurements and alpha angle measurements of arthroscopic hip surgery and physiotherapist-led non-surgical care using independent T-test and one-way ANCOVA

| Parameter | Physiotherapist-led non-surgical care | | | Arthroscopic hip surgery | | | Independent  T-test | One-way ANCOVA |
| --- | --- | --- | --- | --- | --- | --- | --- | --- |
|  | Baseline Mean (SD) | 12 month Mean (SD) | Mean change (SD) | Baseline Mean (SD) | 12 month Mean (SD) | Mean change (SD) | P- value (95% CI) | P- value (95% CI) |
| Total AP anterior coverage (%) | 24.7 (6.26) | 25.4 (5.93) | -1.12 (7.36) | 27.2 (8.16) | 26.7 (5.91) | 0.19 (6.09) | 0.378 (-4.26, 1.64) | 0.823 (-1.881, 2.358) * |
| Total AP posterior coverage (%) | 45.1 (8.30) | 44.8 (8.85) | 0.35 (7.16) | 47.4 (8.15) | 47.0 (8.48) | 0.68 (3.71) | 0.792 (-2.82, 2.16) | 0.890 (-2.247, 2.585) |
| Total femoral head coverage (%) | 81.0 (7.39) | 81.1 (6.83) | -0.32 (6.20) | 82.4 (7.56) | 79.8 (6.72) | 2.38 (8.30) | 0.096 (-5.89, 0.50) | 0.211 (-4.860, 1.092) * |
| LCEA (degrees) | 34.7 (6.68) | 34.0 (5.63) | 0.41 (3.45) | 37.1 (5.41) | 34.2 (6.20) | 2.90 (4.57) | 0.006 (-4.26, -0.72) | **0.030 (-3.403, -0.180) *** |
| Acetabular index (degrees) | 4.66 (4.83) | 5.39 (4.45) | -0.95 (3.82) | 2.52 (4.23) | 4.04 (5.04) | -1.25 (3.63) | 0.720 (-1.33, 1.92) | 0.809 (-1.732, 1.356) |
| ACM-Angle (degrees) | 44.7 (3.56) | 44.6 (3.07) | 0.0095 (2.76) | 45.0 (2.53) | 44.6 (2.84) | 0.28 (2.47) | 0.633 (-1.42, 0.87) | 0.780 (-1.127, 0.849) |
| Extrusion index (%) | 17.0 (5.80) | 17.4 (4.98) | -0.17 (3.12) | 15.0 (4.52) | 18.2 (5.21) | -3.29 (4.47) | 0.000 (1.44, 4.80) | **0.002 (0.882, 3.968) *** |
| Cross-over sign (% agreement) | 0.76 (0.43) | 0.57 (0.50) | 0.19 (0.55) | 0.66 (0.50) | 0.60 (0.50) | 0.024 (0.47) | 0.139 (-0.057, 0.39) | 0.365 (-0.108, 0.290) |
| Retroversion index (%) | 14.8 (14.5) | 11.2 (14.4) | 2.62 (13.8) | 11.3 (12.8) | 9.27 (10.7) | 0.84 (9.89) | 0.498 (-3.45, 7.01) | 0.953 (-4.531, 4.807) |
| Posterior wall sign (% agreement) | 0.73 (0.45) | 0.71 (0.46) | 0.0000 (0.44) | 0.62 (0.49) | 0.64 (0.49) | -0.024 (0.41) | 0.799 (-0.16, 0.21) | 0.854 (-0.182, 0.151) |
| Alpha angle measurement (°) on radially reformatted MRI scans | 69.2 (14.2) | 67.4 (13.5) | 0.34 (2.4) | 70.8 (11.7) | 62.1 (15.9) | 8.8 (11.7) | < 0.001 (-12.08, -4.76) | **< 0.001 (-11.776, -4.772) *** |

*Robust standard errors used
