# Supplementary material for: Do patients with femoroacetabular impingement syndrome who undergo hip arthroscopy display improved alpha angle (magnetic resonance imaging) and radiographic hip morphology?
Source: Int J Rheum Dis. 2022 Dec 11;26(2):354–9. doi: 10.1111/1756-185X.14530 (PMC10946938; doi:10.1111/1756-185X.14530)
Supplement: Supplementary file 4 — Table S4. [file APL-26-354-s003.docx]

**Table 4:** Adjusted comparison of hip parameters of each surgical procedure done using independent T-test, ANOVA and McNemar’s Test

| Parameter | Cam resection only | | | | Acetabular rim trimming only | | | | Both cam resection and acetabular rim trimming | | | | ANOVA |
| --- | --- | --- | --- | --- | --- | --- | --- | --- | --- | --- | --- | --- | --- |
|  | Baseline Mean (SD) | 12 month Mean (SD) | Mean change (SD) | Independent T-test  P-value (95% CI) | Baseline Mean (SD) | 12 month Mean (SD) | Mean change (SD) | Independent T-test  P-value (95% CI) | Baseline Mean (SD) | 12 month Mean (SD) | Mean change (SD) | Independent T-test  P-value (95% CI) |  |
| Total AP anterior coverage (%) | 29.2 (9.71) | 28.1 (6.79) | 1.41 (6.72) | 0.431  (-2.315, 5.128) | 25.8 (7.49) | 24.3 (3.67) | 1.48 (6.13) | 0.618  (-6.128, 9.088) | 25.7 (5.86) | 26.4 (5.86) | -0.71 (5.25) | 0.553  (-3.168, 1.748) | F(2,37)=0.646, p = 0.530 |
| Total AP posterior coverage (%) | 46.9 (6.33) | 48.2 (7.21) | -0.587 (3.53) | 0.530  (-2.541, 1.368) | 58.5 (6.09) | 58.2 (6.07) | 0.300 (0.574) | 0.308  (-0.413, 1.013) | 44.5 (7.89) | 42.7 (7.20) | 1.46 (3.93) | 0.113  (-0.379, 3.299) | F(2,37)=1.430, p = 0.252 |
| Total femoral head coverage (%) | 81.4 (10.6) | 82.2 (7.02) | -1.36 (11.6) | 0.656  (-7.766, 5.046) | 78.9 (1.29) | 77.9 (0.809) | 1.00 (0.900) | 0.068  (-0.118, 2.118) | 83.2 (5.49) | 78.0 (7.03) | 5.73 (5.06) | **< 0.001 (3.360, 8.100)** | **F(2,37)=3.470, p = 0.042** |
| LCEA (degrees) | 37.7 (5.68) | 36.4 (7.05) | 1.73 (5.16) | 0.216  (-1.134, 4.587) | 32.8 (2.23) | 32.4 (4.65) | 0.440 (2.47) | 0.710  (-2.622, 3.502) | 37.0 (5.38) | 32.6 (5.67) | 4.61 (4.19) | **< 0.001 (2.649, 6.571)** | F(2,37)=2.755, p = 0.077 |
| Acetabular index (degrees) | 1.38 (4.20) | 2.45 (4.21) | -1.08 (3.66) | 0.272  (-3.106, 0.946) | 6.70 (4.67) | 6.38 (5.50) | 0.320 (2.12) | 0.752  (-2.310, 2.950) | 2.34 (3.53) | 4.42 (5.57) | -1.90 (4.00) | **0.047**  **(-3.770,**  **-0.0297)** | F(2,37)=0.765, p = 0.473 |
| ACM-Angle (degrees) | 45.5 (2.01) | 44.2 (2.12) | 1.11 (1.06) | **0.001 (0.524, 1.703)** | 44.8 (3.50) | 46.4 (3.98) | -1.56 (6.14) | 0.600  (-9.184, 6.064) | 44.5 (2.69) | 44.5 (3.09) | -0.0750 (1.41) | 0.814  (-0.733, 0.583) | F(2,37)=2.672, p = 0.082 |
| Extrusion index (%) | 14.8 (4.46) | 16.3 (6.13) | -1.77 (5.33) | 0.220  (-4.720, 1.186) | 18.5 (1.37) | 20.1 (3.43) | -1.56 (2.69) | 0.265  (-4.905, 1.785) | 15.1 (4.66) | 19.7 (4.47) | -4.92 (3.81) | **< 0.001**  **(-6.702,**  **-3.138)** | F(2,37)=2.709, p = 0.080 |
| Cross-over sign (% agreement) | 0.710 (0.470) | 0.600 (0.507) | 0.0667 (0.458) |  | 0.400 (0.548) | 0.000 (0.000) | 0.400 (0.548) |  | 0.730 (0.456) | 0.800 (0.410) | -0.100 (0.447) |  | F(2,37)=2.429, p = 0.102 |
| Retroversion index (%) | 11,1 (10.1) | 9.59 (11.4) | -0.0333 (12.3) | 0.992  (-6.821, 6.754) | 3.22 (4.51) | 0.000 (0.000) | 3.22 (4.51) | 0.186  (-2.382, 8.822) | 12.7 (13.9) | 12.3 (10.5) | 0.980 (9.69) | 0.656  (-3.553, 5.513) | F(2,37)=0.187, p = 0.830 |
| Posterior wall sign (% agreement) | 0.650 (0.493) | 0.670 (0.488) | -0.0667 (0.594) |  | 0.000 (0.000) | 0.000 (0.000) | 0.000 (0.000) |  | 0.730 (0.456) | 0.750 (0.444) | 0.0500 (0.224) |  | F(2,37)=0.367, p = 0.695 |
| Alpha angle measurement (°) on radially reformatted MRI scans | 71.5 (9.56) | 59.0 (14.9) | 11.8 (12.6) | **0.003 (4.845, 18.768)** | 72.7 (12.9) | 72.0 (17.7) | 0.64 (8.27) | 0.871 (-9.626, 10.906) | 71.3 (13.1) | 61.8 (16.7) | 9.23 (11.6) | **0.002 (3.938, 14.528)** | F(2,38)=1.713, p = 0.194 |
| McNemar’s test on Cross-over sign (% agreement) | p-value = 1.000 | | | |  | | | | p-value = 0.625 | | | |  |
| McNemar’s test on Posterior wall sign (% agreement) | p-value = 1.000 | | | |  | | | | p-value = 1.000 | | | |  |
