# Supplementary material for: Do patients with femoroacetabular impingement syndrome who undergo hip arthroscopy display improved alpha angle (magnetic resonance imaging) and radiographic hip morphology?
Source: Int J Rheum Dis. 2022 Dec 11;26(2):354–9. doi: 10.1111/1756-185X.14530 (PMC10946938; doi:10.1111/1756-185X.14530)
Supplement: Supplementary file 5 — Table S5. [file APL-26-354-s005.docx]

**Table 5:** Correlation between changes in alpha angle measurements and iHOT-33 scores

| Intervention Group | Pearson Correlation | p-value |
| --- | --- | --- |
| All participants | -0.099 | 0.371 |
| Physiotherapist-led non-surgical care | 0.004 | 0.980 |
| Arthroscopic hip surgery | 0.056 | 0.721 |
