# Supplementary material for: Do patients with femoroacetabular impingement syndrome who undergo hip arthroscopy display improved alpha angle (magnetic resonance imaging) and radiographic hip morphology?
Source: Int J Rheum Dis. 2022 Dec 11;26(2):354–9. doi: 10.1111/1756-185X.14530 (PMC10946938; doi:10.1111/1756-185X.14530)
Supplement: Supplementary file 6 — Figure S1. [file APL-26-354-s001.docx]

**Supplementary Figure**


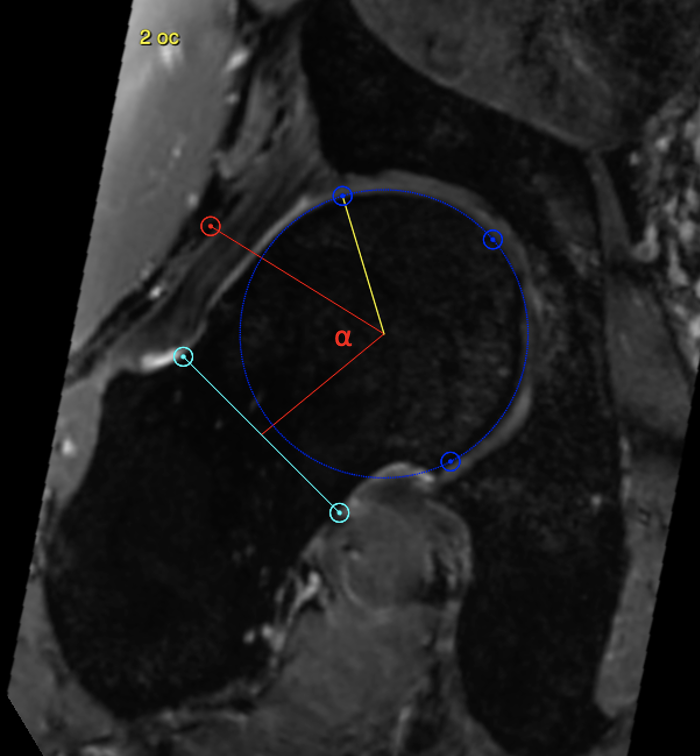


**Figure 1:** Alpha angle was measured in each reconstructed radial plane using Orthopedic Studio OsiriX plugin Version 1.3.3b (Carl Siversson, Lund University, Sweden).

Legend: The alpha angle was measured using a line formed between the center of the femoral head and the point where the distance from the femoral head center to the peripheral contour of the femoral head exceeds the radius of the femoral head and a second line in the axis of the femoral neck.^1^

**References**

1. Sutter R, Dietrich TJ, Zingg PO, et al. How Useful Is the Alpha Angle for Discriminating between Symptomatic Patients with Cam-type Femoroacetabular Impingement and Asymptomatic Volunteers? *Radiology* 2012; 264: 514-521. DOI: 10.1148/radiol.12112479.
